# Author Correction: Evaluating Tumor Evolution via Genomic Profiling of Individual Tumor Spheroids in a Malignant Ascites

**DOI:** 10.1038/s41598-018-35526-w

**Published:** 2018-11-21

**Authors:** Sungsik Kim, Soochi Kim, Jinhyun Kim, Boyun Kim, Se Ik Kim, Min A. Kim, Sunghoon Kwon, Yong Sang Song

**Affiliations:** 1 Department of Electrical and Computer Engineering, Seoul National University, Seoul, 08826 Republic of Korea; 2 Institutes of Entrepreneurial BioConvergence, Seoul National University, Seoul, 08826 Republic of Korea; 3 Seoul National University Hospital Biomedical Research Institute, Seoul National University Hospital, Seoul, 03080 Republic of Korea; 4 Interdisciplinary Program for Bioengineering, Seoul National University, Seoul, 08826 Republic of Korea; 5 Cancer Research Institute, Seoul National University College of Medicine, Seoul, 03080 Republic of Korea; 6 Department of Anesthesiology, McGovern Medical School, The University of Texas Health Science Center at Houston, Houston, TX USA; 7 Department of Obstetrics and Gynecology, Seoul National University College of Medicine, Seoul, 03080 Republic of Korea; 8 Department of Pathology, Seoul National University College of Medicine, Seoul, 03080 Republic of Korea; 9 Interdisciplinary Program in Cancer Biology, Seoul National University College of Medicine, Seoul, 03080 Republic of Korea; 10 Biomodulation Department of Agricultural Biotechnology, Seoul National University, Seoul, 03080 Republic of Korea

Correction to: *Scientific Reports* 10.1038/s41598-018-31097-y, published online 24 August 2018

The Acknowledgements section in this Article is incomplete.

“This research was supported by a grant of the Korea Health Technology R&D Project through the Korea Health Industry Development R&D Project through the Korea Health Industry Development Institute (KHIDI), funded by the Ministry of Health & Welfare, Republic of Korea (grant number: HI16C2037 and HA17C0037).”

should read:

“This research was supported by a grant of the Korea Health Technology R&D Project through the Korea Health Industry Development R&D Project through the Korea Health Industry Development Institute (KHIDI), funded by the Ministry of Health & Welfare, Republic of Korea (grant number: HI16C2037 and HA17C0037). This research was also supported by Global Research Development Center Program through the National Research Foundation of Korea (NRF) funded by the Ministry of Science and ICT (MSIT) (2015K1A4A3047345).”

