# Supplementary material for: Evaluating Tumor Evolution via Genomic Profiling of Individual Tumor Spheroids in a Malignant Ascites
Source: Sci Rep. 2018 Aug 24;8:12724. doi: 10.1038/s41598-018-31097-y (PMC6109089; doi:10.1038/s41598-018-31097-y)
Supplement: Supplementary file 1 — Supplementary Figures [file 41598_2018_31097_MOESM1_ESM.docx]

## **Evaluating Tumor Evolution via Genomic Profiling of Individual Tumor Spheroids in a Malignant Ascites**

Sungsik Kim^1, 4, 11^, Soochi Kim^3, 5, 11^, Jinhyun Kim^1^, Boyun Kim^6^, Se Ik Kim^7^, Min A Kim^8^, Sunghoon Kwon^1-3, *^, and Yong Sang Song^3, 5, 7, 9, 10 *^

^1^ Department of Electrical and Computer Engineering, Seoul National University, Seoul 08826,

Republic of Korea

^2^ Institutes of Entrepreneurial BioConvergence, Seoul National University, Seoul 08826,

Republic of Korea

^3^ Seoul National University Hospital Biomedical Research Institute, Seoul National University Hospital, Seoul 03080, Republic of Korea

^4^ Interdisciplinary Program for Bioengineering, Seoul National University, Seoul 08826, Republic of Korea

^5^ Cancer Research Institute, Seoul National University College of Medicine, Seoul 03080, Republic of Korea

^6^ Department of Anesthesiology, McGovern Medical School, The University of Texas Health Science Center at Houston, Houston, TX, USA

^7^ Department of Obstetrics and Gynecology, Seoul National University College of Medicine, Seoul 03080, Republic of Korea

^8^ Department of Pathology, Seoul National University College of Medicine, Seoul 03080, Republic of Korea

^9^ Interdisciplinary Program in Cancer Biology, Seoul National University College of Medicine, Seoul 03080, Republic of Korea

^10^ Biomodulation Department of Agricultural Biotechnology, Seoul National University, Seoul 03080, Republic of Korea

^11^ These authors contributed equally to this work

^*^ These authors jointly supervised this work. Correspondence should be addressed to SH.K. (skwon@snu.ac.kr) or Y.S.S (yssong@snu.ac.kr)

Supplementary Fig. S1

**Overall process for detecting single nucleotide variants**

**
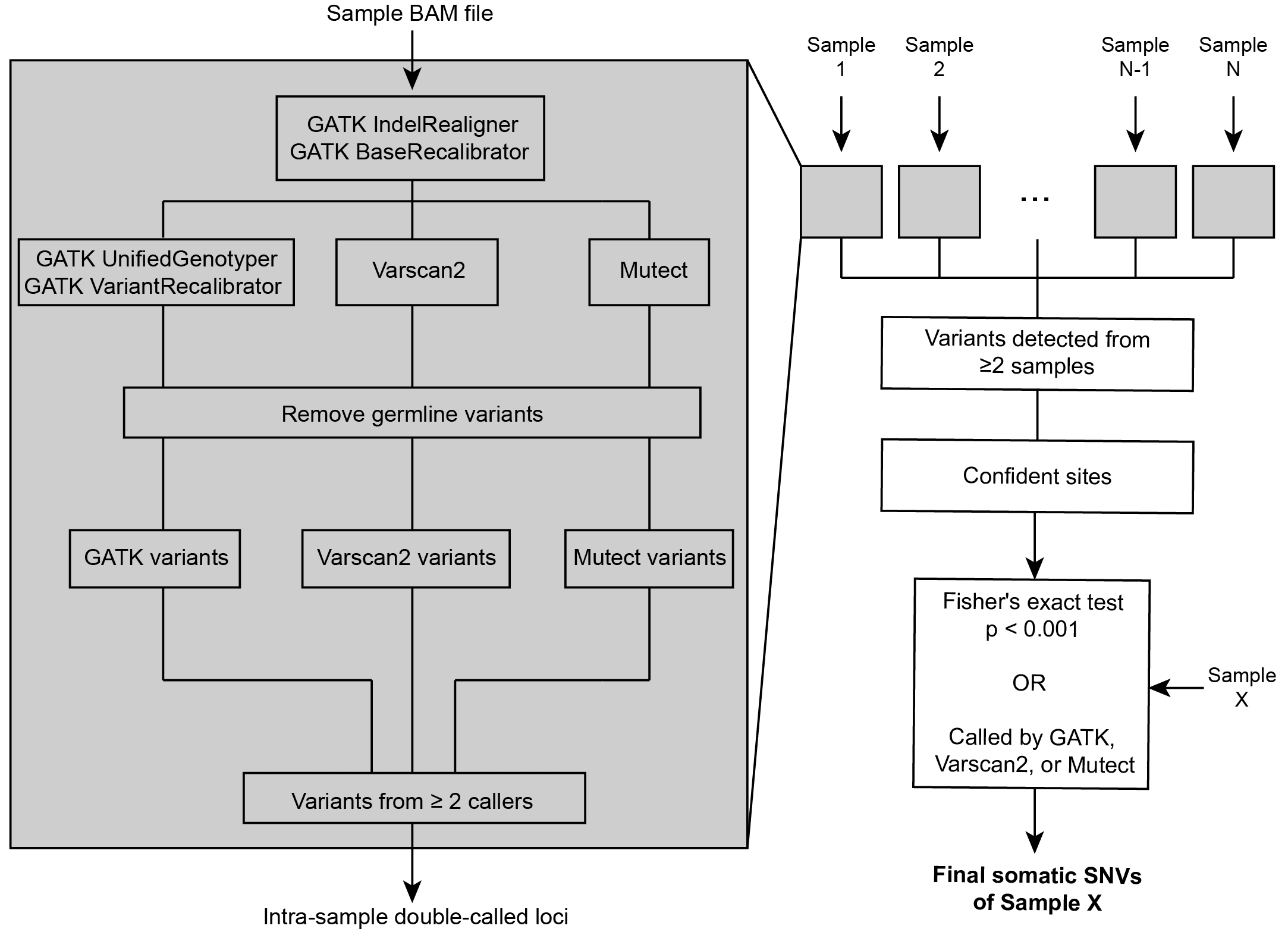
**

Supplementary Fig. S2

**Variant allele frequencies according to the occurrence of the variants**

For each group of (**A**) Primary clone and (**B**) Ascites clone 1, the variant allele frequencies were plotted according to the occurrence of the variants in each group. In the case of Primary clone, the allele frequencies tend to increase as the occurrence of variants in each group increases (Kruskal-Wallis rank sum test, p < 0.01). This could suggest that cells with the mutations which were occurred later in the evolutionary process tend to occupy a small percentage of all primary cancer cells. However, this tendency was not shown in Ascites Clone 1 (Kruskal-Wallis rank sum test, p = 0.07). This result can be interpreted to indicate that, compared with the primary tissue samples, each tumor spheroid was comprised of genetically homogeneous tumor cells. The solid lines represent the mean of the allele frequencies.

**
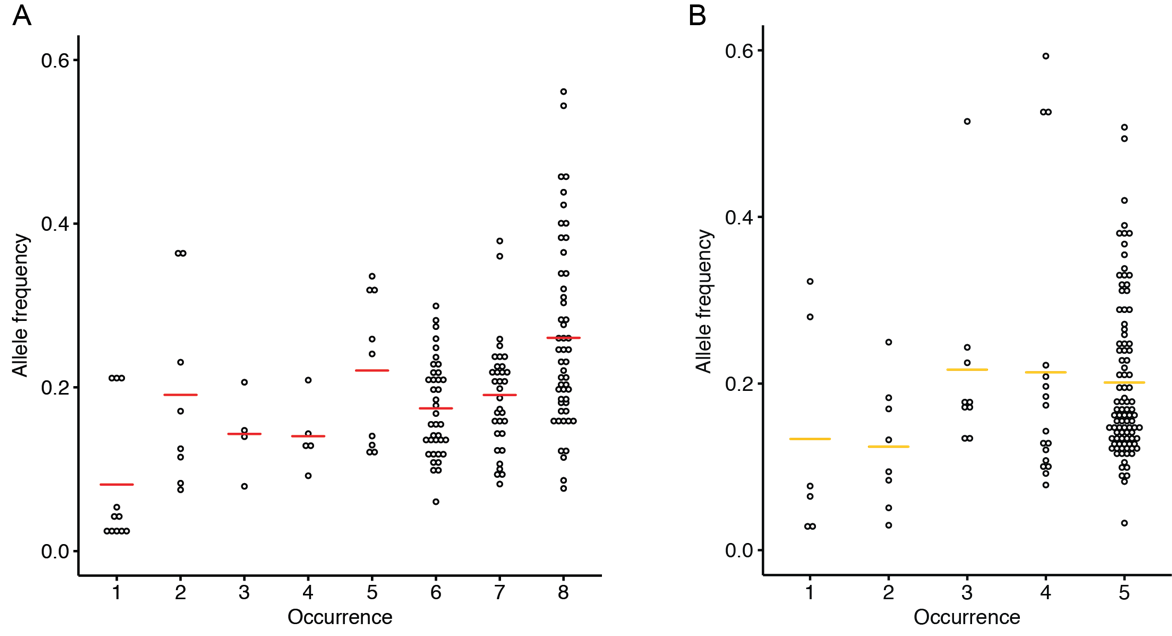
**

Supplementary Fig. S3

**Circos plot of the major subclones**

The Circos plot presents the genome-wide alterations in the ancestral, Primary, and Ascites subclones. For the SNVs, the black, red, and yellow bars represent the ancestral, primary-only, and ascites-only mutations, respectively. The tumor cells acquired the ancestral mutations before dividing into the Primary and Ascites clones. After division, the Primary and Ascites clones acquired lineage-specific SNVs. For the CNAs, the red and blue bars represent amplification and deletion, respectively.

**
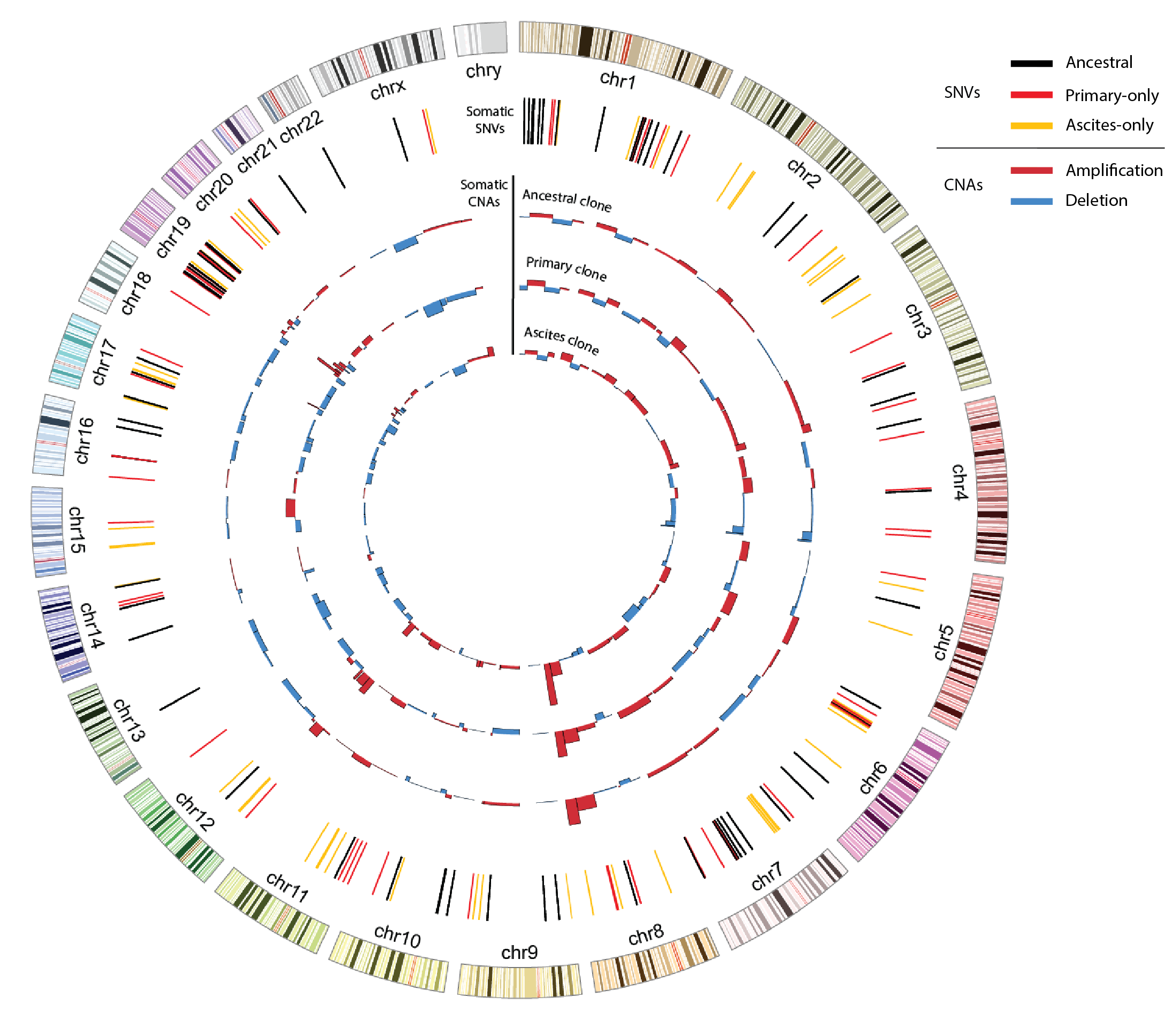
**
